# Developing a multivariable prediction model of global health-related quality of life in patients treated for rectal cancer: a prospective study in five countries

**DOI:** 10.1007/s00384-024-04605-y

**Published:** 2024-03-05

**Authors:** John Andersson, Eva Angenete, Martin Gellerstedt, Eva Haglind

**Affiliations:** 1 Department of General and Orthopaedic Surgery, Alingsås Hospital, Alingsås, Sweden; 2 Department of Surgery, SSORG - Scandinavian Surgical Outcomes Research Group, Institute of Clinical Sciences, Sahlgrenska Academy, University of Gothenburg, Gothenburg, Sweden; 3 Department of Surgery, Region Västra Götaland, Sahlgrenska University Hospital/Östra, Gothenburg, Sweden; 4 School of Health Sciences, University of Skövde, Skövde, Sweden

**Keywords:** Colorectal cancer, Quality of life, Risk factors, EORTC QLQ, Fatigue, Pain

## Abstract

**Purpose:**

Rectal cancer and its treatment have a negative impact on health-related quality of life (HRQoL). If risk factors for sustained low HRQoL could be identified early, ideally before the start of treatment, individualised interventions could be identified and implemented to maintain or improve HRQoL. The study aimed to develop a multivariable prediction model for global HRQoL 12 months after rectal cancer treatment.

**Methods:**

Within COLOR II, a randomised, multicentre, international trial of laparoscopic and open surgery for rectal cancer, a sub-study on HRQoL included 385 patients in 12 hospitals and five countries. The HRQoL study was optional for hospitals in the COLOR II trial. EORTC QLQ-C30 and EORTC QLQ-CR38 were analysed preoperatively and at 1 and 12 months postoperatively. In exploratory analyses, correlations between age, sex, fatigue, pain, ASA classification, complications, and symptoms after surgery to HRQoL were studied. Bivariate initial analyses were followed by multivariate regression models.

**Results:**

Patient characteristics and clinical factors explained 4–10% of the variation in global HRQoL. The patient-reported outcomes from EORTC QLQ-C30 explained 55–65% of the variation in global HRQoL. The predominant predictors were fatigue and pain, which significantly impacted global HRQoL at all time points measured.

**Conclusion:**

We found that fatigue and pain were two significant factors associated with posttreatment global HRQoL in patients treated for rectal cancer T1-T3 Nx. Interventions to reduce fatigue and pain could enhance global HRQoL after rectal cancer treatment.

**Trial registration:**

This trial is registered with ClinicalTrials.gov No. NCT00297791

**Supplementary Information:**

The online version contains supplementary material available at 10.1007/s00384-024-04605-y.

## Background

Local recurrence and survival outcomes after curative treatment for rectal cancer, surgery with/without (chemo)radiotherapy, have improved over the last 20 years [1, 2]. Surgery is still considered a keystone for curative treatment, but for a minority of patients, chemoradiation may result in a complete response. However, the safety of treatment with chemoradiation alone needs further evidence before implementation [3, 4].

Rectal cancer and its treatment can affect patient-reported bodily functions and health-related quality of life (HRQoL) [5–8]. About one third of patients receive a permanent stoma, which may affect HRQoL [9]. Since the tumour is situated in the pelvis, faecal, sexual, and urinary dysfunctions are relatively common after treatment [10, 11]. Patient-reported health-related quality of life (HRQoL) is accepted as an important endpoint in clinical trials [12]. HRQoL is often measured by validated questionnaires, for example, EuroQol-5D (EQ-5D), RAND-Short form 36 (SF-36), and instruments from the European Organisation of Research and Treatment of Cancer (EORTC). Most instruments include the domains of physical, social, and emotional/psychological functioning as well as several symptom scales [13].

A comparison between patients with colon cancer in the COlorectal cancer Laparoscopic or Open Resection (COLOR) trial and patients with rectal cancer in the Colorectal Cancer Laparoscopic or Open Resection COLOR II trial found that the impact of treatment on HRQoL, as measured by EORTC Quality of Life-Core 30 (QLQ-C30), was more profound and longer lasting in rectal cancer patients [14, 15]. As there are many long-term survivors after treatment for rectal cancer, the need to evaluate long-term treatment effects on QoL has increased (1–2) but also to attempt to discover predictors of continued low HRQoL. Identifying predictors is a basis for studies on interventions aimed at improving HRQOL.

This study aimed to develop a model of predictors for global HRQoL 12 months after rectal cancer treatment, among treatment factors, domains, or symptoms. We also examined changes in these variables postoperatively (1 month) and long term (12 months) after surgery.

## Methods

### Source of data—clinical trial

COlorectal cancer Laparoscopic or Open COLOR II was an international, randomised non-inferiority clinical trial in which 30 hospitals in eight countries took part. The primary aim was to compare local recurrence after laparoscopic and open surgery as treatment of rectal cancer, respectively, and patients were recruited between 2004 and 2010 [16]. The ratio of randomisation between laparoscopic and open surgery was two to one. Follow-up was yearly during 5 years.

### Participants—clinical trial

Patients with rectal cancer within 15 cm from the anal verge, no metastases, and planned surgery with curative intent were eligible. Patients with tumours invading adjacent tissues or organs, T4 tumours, or T3 rectal cancers within 2 mm of the endopelvic fascia at preoperative workup were excluded. Further details about inclusion/exclusion criteria, randomisation, and short- and long-term clinical outcomes have been reported elsewhere [16, 17]. Annual clinical assessments were carried out for 5 years according to the trial protocol and reported in a standardised clinical record form.

### Source of data—HRQoL study within clinical trial

In this explorative study, we used data for the cohort participating in the HRQoL study within the clinical trial COLOR II (15) to develop a model of predictors. Participation in the HRQoL study was optional at both the centre and individual level. Twelve hospitals in Canada, Denmark, Germany, the Netherlands, and Sweden opted to participate in the HRQL component of the COLOR II trial.

### Participants—HRQoL study

All participants were part of the COLOR II randomised trial. Inclusion criteria for the HRQoL study were ability to understand the questionnaires, and informed consent after verbal and written information had been given with opportunities for questions.

### Data collection and questionnaires

Data on demographics, clinical details, complications, and more was collected from the database for COLOR II trial. HRQoL was measured using EORTC QLQ-C30 and EORTC Quality of Life-Colorectal 38 (QLQ-CR38) validated questionnaires in Swedish, Dutch, Danish, English, and German translations [18, 19], as well as EuroQol 5D-3L (15). Patient-reported HRQoL was collected preoperatively and 1, 6, 12, and 24 months postoperatively. In this study, we used data collected with the two EORTC instruments preoperatively and at 1 and 12 months postoperatively. Administration of questionnaires was by the research personnel at each centre (baseline) and by post for later follow-up points; return was by post in prepaid envelopes.

EORTC QLQ-C30 is a widely used validated instrument in cancer, which examines patient-reported HRQoL. It consists of 30 questions, which cover five functional domains (physical, role, social, cognitive, and emotional functioning), three symptom scales (pain, fatigue, and nausea/vomiting), six item scales of problems common in patients with cancer (dyspnoea, insomnia, loss of appetite, constipation, diarrhoea, and financial difficulties), and finally, a global health/QoL index—hereafter, global HRQoL.

EORTC QLQ-CR38 is a disease-specific validated supplement to EORTC QLQ-C30 for patients with colorectal cancer and consists of 38 additional questions. These questions cover four functional scales/single items (body image, sexual functioning, sexual enjoyment, future perspective) and eight symptom scales/items (micturition problems, chemotherapy side effects, symptoms related to the gastrointestinal tract, male sexual problems, female sexual problems, defecation problems, stoma-related problems, and weight loss). EORTC QLQ-CR38 has since been revised into EORTC QLQ-CR29 [20, 21].

### Outcomes

To identify factors present at diagnosis of rectal cancer that predicted HRQoL 12 months after surgery by using EORTC instruments. Blinding was not practised.

### Sample size

The HRQoL, symptoms, and functions were secondary outcomes in COLOR II, and thus no sample size calculations were made for this outcome. However, for the clinical trial as such, sample size calculations were made for the primary outcome “local recurrence” where results in the two randomisation arms were compared using a non-inferiority design (17).

### Missing data

No imputations of missing values were performed. Complete case analyses were made.

### Ethics

The appropriate ethical authorities gave consent to the trial including the HRQoL study in each participating country.

COLOR II was registered at ClinicalTrials.gov (NCT0029779).

### Statistical analysis

EORTC QLQ-C30 and EORTC QLQ-CR38 data were analysed according to the respective scoring manuals and expressed as scores ranging from 0 to 100. In a functional scale, a high score corresponds to a high level of functioning, while a high value in a symptom scale corresponds to a high level of symptoms. EORTC QLQ-CR38 questions regarding defecation problems were exclusively answered by patients without a stoma, while questions regarding stoma-related problems were only answered by patients with a stoma. The two symptom scales were merged into one in the analyses.

### Predictors

All domains, symptom scales, and items were treated as continuous variables. American Society of Anaesthesiologists (ASA) classification was dichotomised as no disease (ASA 1) versus ASA 2–4. Tumour class was dichotomised as T1 and T2 versus T3. Complications were analysed as classified in the clinical record form.

### Statistical models

First, we examined the potential relationships between global QoL and patient factors, treatment factors, functional domains, and symptom scales in the EORTC QLQ-C30 and EORTC QLQ-CR38 questionnaires by bivariate analysis at each time point. Student *t*-test, Spearman rank correlation test, Pearson’s correlation test, and ANOVA were used when appropriate depending on the type of variable. These analyses were used for selecting variables to enter into a multiple regression model. In this pre-selection process, we used a *p*-value of 0.20.

Second, all factors with a *p*-value < 0.20 were included in four multiple linear regression models: (1) patient characteristics and treatment factors, (2) complications within the postoperative period and at the 1-year follow-up, (3) EORTC QLQ-C30 functioning scales and symptom, and (4) EORTC QLQ-CR38 functioning domains and symptoms. The significance level was set to *p* < 0.05. All models were adjusted for baseline, i.e. preoperative, global QoL. Finally, a multivariable model including significant variables from the four models was analysed at each of the three time points. The final multivariable analyses were adjusted for baseline global QoL. Significant factors (*p* < 0.05) in these analyses constitute the final results. Validation was not performed.

### Assessment of model performance

Multiple correlation coefficient, i.e. determination coefficient *R*^2^.

All statistical calculations were performed in IBMSPSS (SPSS® 22 software, IBM, Armonk, NY, USA).

## Results

**Fig. 1:**
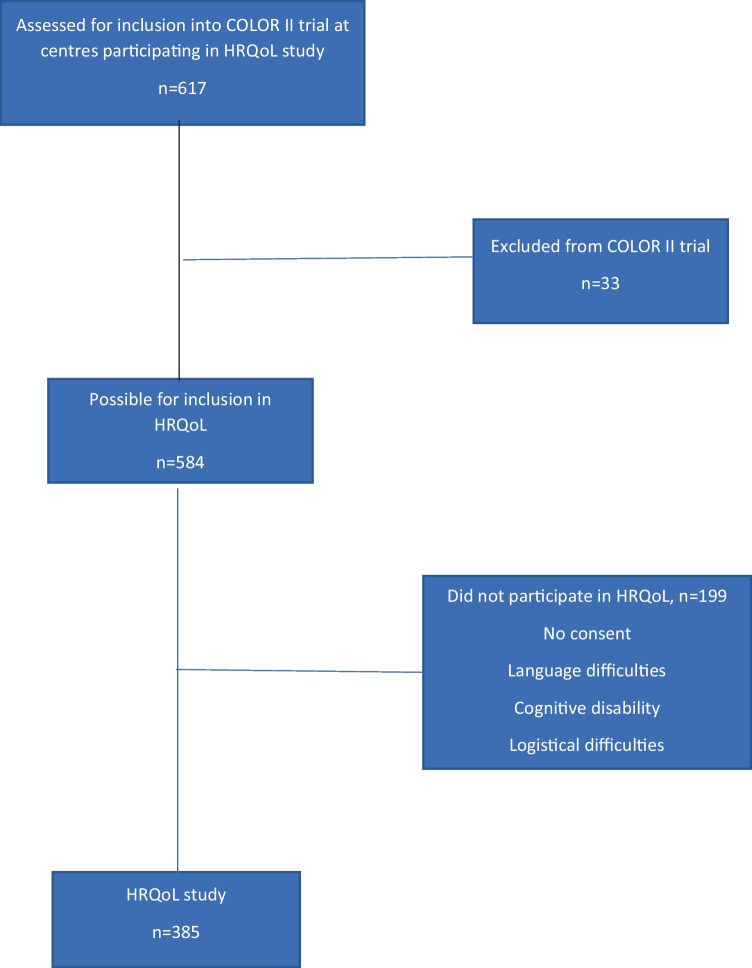
Flow chart

Out of 30 hospitals recruiting patients to COLOR II, 12 hospitals participated and included a total of 385 in the HRQL study (Fig. 1), and this constitutes the cohort for the current analysis.

Baseline patient characteristics and treatment factors are presented in Table 1. Response rates to questionnaires varied between 91% at baseline and 79% at 12 months, with a percentage of missing for EORTC QLQ-C30 at baseline at 8.6%, at 1 month at 12.2%, and at 12 months at 20.8% and for EORTC QLQ-CR38; the corresponding figures were 9.3%, 11.4%, and 20.5%.

**Table 1:** Patient and treatment characteristics (*n* = 385)

**Patient and treatment characteristics**	
Sex, *n* (%)	
Male	239 (62)
Female	146 (38)
Age, years (CI)	67.1 (66.1–68.1*)
BMI, kg/m^2^ (CI)	26 (25.6–26.5*)
ASA classification, *n* (%)	
1	103 (27)
2	224 (58)
3	55 (14)
4	2 (1)
Missing	1 (0.3)
Tumour stage, *n* (%)	
I	22 (6)
II	135 (35)
III	207 (54)
IV	12 (3)
Missing	9 (2)
Type of resection, *n* (%)	
PME	42 (11)
TME	220 (57)
APE	116 (30)
Other	5 (1)
Missing	2 (1)
Preop radiotherapy, *n* (%)	
Yes	216 (56)
No	168 (44)
Missing	1 (0.3)
Preop chemotherapy, *n* (%)	
Yes	64 (17)
No	290 (75)
Missing	31 (8)
Blood loss, ml (CI)	463 (404–522*)
Skin-to-skin time, min (CI)	253 (244–262*)
Conversion, *n* (%)	67 (17)

Classification of tumour stage corresponds to the pathology report of the resected specimen

*ASA* American Society of Anaesthesiologists, *BMI* body mass index, *CI* confidence interval, *PME* partial mesorectal excision, *TME* total mesorectal excision, *APE* abdominoperineal excision

*Values are mean with 95% CI

Patient characteristics as well as data on treatment to describe the cohort are detailed in Table 1. In comparison with the entire cohort in the trial COLOR II, there were no obvious differences in sex, age, body mass index, ASA category, preoperative radiotherapy, type of surgery performed, or conversion from laparoscopic technique [16]. Clinical tumour stage differed with a lower percentage in this study having stage I and a lower percentage had preoperative chemotherapy [16].

Complications within the 1 month (postoperative period) and at the 1-year follow-up are presented in Table 2. In comparison with the entire COLOR II trial results at 1 month, fewer patients with an anastomotic leak answered the questionnaires, but apart from this, the group participating in the HRQoL study was comparable [16].

**Table 2:** Complications within the 1 month and at the 1-year follow-up

**Postoperative complications**	***n*** **(%)**
Any complication	132 (34)
Anastomotic leakage	17 (4)
Cardiac	14 (4)
Respiratory	11 (3)
Abscess	19 (5)
Ileus	10 (3)
Other	102 (27)
Re-intervention	49 (13)
Readmission	36 (9)

**Complications at the 1-year follow-up**	***n* (%)**
Any complication	114 (30)
Incisional hernia	16 (4)
Bowel function	23 (6)
Stress urinary incontinence	15 (4)
Sexual dysfunction	28 (7)
Faecal incontinence	15 (4)
Fistula	2 (1)
Perineal hernia	6 (2)
Parastomal hernia	9 (2)
Perineal wound defect	3 (1)
Anastomotic leakage/presacral abscess	9 (2)
Pain	5 (1)
Other	53 (14)
Re-intervention	83 (22)
Readmission	151 (40)
Recurrence	51 (13)
Locoregional recurrence	10 (3)

### Pre-selection—bivariate analyses

Analysing predictive factors among patient characteristics and treatment-related factors, ASA classification and length of hospital stay were found to be associated (bivariate analyses, *p* < 0.20) with global HRQoL, at all time points. Regarding body mass index (BMI), age and preoperative (chemo)radiotherapy, tumour (T)-classification, node status (N-status), and type of resection showed a varied pattern regarding significant association with global HRQoL over time. Male/female was not (*p* > 0.20) a predictive factor. Analysing factors within 1 month of surgery, that is complications, as predictors of global HRQoL, the bivariate analysis revealed that all complications except cardiac, respiratory, and readmission showed significant associations. In the analysis of long-term events within 12 months, complications, readmission, and recurrence were significantly associated with global HRQoL.

EORTC QLQ-C30 domains were analysed as possible predictors, and functioning and symptom scales were significantly associated with global HRQoL (*p* < 0.20) in the bivariate analyses at all time points except for constipation and financial difficulties, which varied over time. EORTC QLQ-CR38 functioning and symptom scales all significantly influenced global HRQoL (*p* < 0.20) at all time points except sexual enjoyment and male sexual problems, which showed a varied pattern.

### Multivariable models

Analysing patient characteristics and complications in multivariate models, few factors were significantly associated with global HRQoL. The degree of explanation (*R*^2^ values) showed that 4–10% of global HRQoL was explained by these factors. After adjusting for baseline global QoL, *R*^2^ increased to 30% at 12 months (Supplementary Table 1).

Other domains, functioning, and symptom scales of EORTC QLQ-C30 explained 55–65% of global HRQoL with the strongest associations 1 month after surgery (Supplementary Table 2).

The models of EORTC QLQ-CR38 and global HRQoL correlations (*R*^2^) could explain 42–55% of global QoL (Supplementary Table 3).

The final models revealed fatigue and pain as significantly associated with global HRQoL, preoperatively, at 1 month and 12 months (Table 3).

**Table 3:** Final trimmed models, analysing significant factors from bivariate models versus global HRQoL

	**Preoperative** ***R***^**2**^** = 0.55**		**1 month** ***R***^**2**^** = 0.64**		**12 months** ***R***^**2**^** = 0.56**	
**Variable**	**Coef**	***p***	**Coef**	***p***	**Coef**	***p***
Baseline global QoL	-	-	0.09	0.023	0.24	< 0.001
Patient and treatment factors						
Age	-	-	-	-	− 0.21	0.008
Complications						
Postoperative ileus	-	-	− 10.1	0.026	-	-
Recurrence	-	-	-	-	− 5.8	0.012
EORTC QLQ-C30^a^ (missing)	(33/385)		(47/385)		(80/385)	
Physical function	0.27	< 0.001	-	-	-	-
Emotional function	0.19	< 0.001	0.13	0.008	-	-
Social function	-	-	0.14	< 0.001	0.21	< 0.001
Fatigue	− 0.19	< 0.001	− 0.23	< 0.001	− 0.27	< 0.001
Nausea and vomiting	-	-	− 0.14	0.015	-	-
Pain	− 0.13	0.005	− 0.14	< 0.001	− 0.18	0.001
Insomnia	− 0.08	0.014	-	-	-	-
Appetite loss	-	-	− 0.07	0.015	-	-
Diarrhoea	− 0.09	0.001	-	-	-	-
EORTC QLQ-CR38^b^ (missing)	(36/385)		(44/385)		(79/385)	
Future perspective	0.07	0.021	-	-	-	-

^a^*EORTC QLQ-C30* European Organisation of Research and Treatment of Cancer Quality of Life—Core 30

^b^*EORTC QLQ-CR38* European Organisation of Research and Treatment of Cancer Quality of Life—Colorectal 38

No other factors were significantly associated with global HRQoL at all three time points (Supplementary Table 3).

Before surgery, fatigue and pain as well as physical and emotional function, insomnia, diarrhoea, and future perspective were associated with global HRQoL. One month after surgery, fatigue and pain were still of significantly associated with global HRQoL together with emotional and social function, nausea, vomiting, and appetite loss. Twelve months after surgery, the pattern of factors significantly associated with global HRQoL again included fatigue and pain, as well as social function. Of the common clinical factors, postoperative ileus was associated with global HRQoL 1 month after surgery. Age and recurrence correlated significantly with global HRQoL after 12 months.

## Discussion

Our analysis of HRQoL suggested that fatigue and pain in the EORTC QLQ-C30 questionnaire were significantly associated with global HRQoL in patients with potentially curable rectal cancer, both before and after surgery.

We previously reported compliance and outcomes using EORTC QLQ-C30 and EORTC QLQ-CR38 in this cohort, including bodily functions [11, 15]. Our present work suggests that fatigue is an important factor for global HRQoL in patients undergoing curative treatment for rectal cancer. Previous studies in patients with metastasised cancer and breast cancer have reported fatigue as a major contributor to low global HRQoL [22, 23].

The emotional function domain in EORTC QLQ-C30 has been shown to predominantly assess anxiety rather than depression. However, in a previous study, depression was a stronger predictor of low HRQoL than anxiety using a combination of the Hospital Depression and Anxiety Scale (HADS) and the EORTC QLQ-C30 instrument [24]. Another study using the same instruments reported that depression, as well as anxiety, were associated with lower global HRQoL [25]. In our study, we did not measure depression and anxiety by HADS, which could in part explain, why our results differ from what has been reported earlier.

In our analysis, emotional functioning was associated with global HRQoL preoperatively and at 1 month after surgery, but not at 12 months. We suggest that this may have been due to a response shift during the path of treatment as patients adapted to their new situation [26]. Before surgery (baseline), patients had received a cancer diagnosis and a treatment plan. They then underwent (multimodal) treatment with a risk of morbidity and may have been uncertain of cure. This may have affected emotional function, insomnia, fatigue, and pain, which could subsequently influence global HRQoL. One month after surgery, most patients were not fully recovered, and some anticipated adjuvant chemotherapy with probable morbidity. These combined effects could have been reflected in the domains and symptom scales, such as nausea, vomiting, and appetite loss. At this time, patients may have also become aware of their social difficulties.

On the other hand, 12 months after surgery, most patients had finished their adjuvant treatment and recovered but may still have been affected by the long-term morbidity of the combined treatment. Fatigue has been considered a difficult-to-treat symptom. Trials are underway in other types of cancer to improve the symptom cluster of fatigue, pain, depression, and anxiety using cognitive behavioural therapy [27, 28]. Recently, randomised trials and meta-analyses have reported possible positive effects on fatigue by physical activity among patients with colorectal cancer and survivors after different types of cancer [29–31].

In our cohort, fixed patient-related factors, such as age, were not as important as modifiable factors, such as pain or urinary dysfunction. This is in accordance with earlier work and should encourage efforts to counteract modifiable risk factors through interventions [32]. Clinical variables affecting global HRQoL are not easy to modify, but consistent efforts to reduce recurrence have improved long-term outcomes. In particular, postoperative ileus has been addressed by systematic efforts for early mobilisation, and bowel obstruction has been improved over time through the increased use of laparoscopic surgery [33–35].

In our analysis, the degree of explanation (*R*^2^) was relatively high when EORTC QLQ-CR38 sub-items were analysed as predictive for global HRQoL, compared with the degree of explanation when, for example, postoperative complications were tested as predictive factors. This could partly be explained by a cognitive bias (halo effect) as global HRQoL was addressed at the end of the same questionnaire as the sub-items [36]. Further research could examine how the relationship between sub-items and global HRQoL changes in response to the order of the questions, e.g. if the global HRQoL question is asked before the sub-items or if questions about postoperative complications are added to the same questionnaire. It would also be of interest to study *R*^2^ longitudinally to see if global HRQoL is equally explained by EORTC QLQ-CR38 sub-items over time. We adjusted for baseline global HRQoL in all models as this has been reported as the most important predictive factor [22]. In our analysis, the impact of baseline global HRQoL increased over time, with the highest association at 12 months after surgery. This may be due to patients’ assessment of HRQoL being influenced by personality factors [37] and individual coping strategies introduced when the patient received his/her cancer diagnosis [31, 38, 39].

Our findings of the impact of pain and fatigue symptoms on global HRQoL should intensify attempts to identify patients with these problems before surgery. Most preoperative risk assessments have focused on physical health problems. It should be noted that an association between severe low anterior resection syndrome (LARS) and low HRQoL measured by EORTC QLQ-C30 has been reported [40]. However, the LARS instrument was first described towards the end of the inclusion period in the COLOR II trial and, thus, was not an instrument used in this study. Nevertheless, assessment of bowel function was part of the EORTC instruments [15].

### Limitations and strengths

One limitation of this study is its explorative nature. Results should be interpreted cautiously, as validation of the model was not performed. A further limitation is that participation in the HRQoL study was optional for centres and patients participating in the COLOR II trial. As described previously, there was a selection bias in the cohort with somewhat healthier participants (ASA classification) in the HRQoL study than the entire COLOR II trial population which to some degree could affect possible generalisations [15].

The major strength of this study was the use of validated questionnaires administered before (baseline) and after surgery at predetermined time points, thus minimising the risk of recall bias. Furthermore, questionnaires were returned to the trial secretariat, a third party [41], reducing the risk of bias. Also, our homogenous cohort, consisting of newly diagnosed patients with potentially curable rectal cancer, should reduce variability and make the outcomes clinically useful. In all 12 centres from five countries participated, the rate of patient participation was 66% of the eligible. Compliance (answering questionnaires) among participants was high at all three time-points.

### Interpretation and clinical implications

Fatigue and pain before the start of treatment was significantly associated with patient-reported global HRQoL also at 12 months after treatment among patients with potentially curable rectal cancer. Our findings suggest that patients with these symptoms could be identified at diagnosis.

Physical activity has been evaluated in several studies and trials with effects on fatigue, also with a sustainable impact on global HRQoL. Another possible intervention against fatigue is cognitive behavioural therapy. However, both suggested interventions need further evidence of efficacy and importantly any clinical implementation should be in a form possible to evaluate.

## Conclusions

A multivariable model of predictors of postoperative HRQoL was developed revealing baseline fatigue and pain in patients with rectal cancer as significantly associated with global HRQoL postoperatively. Clinical interventions aiming to decrease fatigue and pain at baseline could improve global HRQoL but need further evaluation.

## Supplementary Information

Below is the link to the electronic supplementary material.

Supplementary file1 (PDF 179 KB)

## Data Availability

Due to the sensitivity of the data and restrictions in some of the ethical permissions, original data cannot be made available.
