# Supplementary material for: Developing a multivariable prediction model of global health-related quality of life in patients treated for rectal cancer: a prospective study in five countries
Source: Int J Colorectal Dis. 2024 Mar 5;39(1):35. doi: 10.1007/s00384-024-04605-y (PMC10914847; doi:10.1007/s00384-024-04605-y)
Supplement: Supplementary file 1 — Supplementary file1 (PDF 179 KB) [file 384_2024_4605_MOESM1_ESM.pdf]

Developing a multivariable prediction model of global health related quality of life in patients treated for rectal cancer. A prospective study in five countries.

Int J Colorectal Disease

John Andersson[1,2], Eva Angenete[2,3], Ulf Angerås[2], Martin Gellerstedt[4] Eva Haglind[2,3]

[1] Department of General and Orthopaedic Surgery, Alingsås Hospital, Alingsås.

[2] Department of Surgery, SSORG - Scandinavian Surgical Outcomes Research Group, Institute of Clinical Sciences, Sahlgrenska Academy, University of Gothenburg, Gothenburg, Sweden

[3] Region Västra Götaland, Sahlgrenska University Hospital/Östra, Department of Surgery, Gothenburg, Sweden

[4] School of Health Sciences, University of Skövde, Skövde, Sweden.

Corresponding author: Eva Haglind, MD, PhD, eva.haglind@vgregion.se

Supplement Table 1. Multivariate models of global HRQoL in the EORTC QLQ-C30 questionnaire, patient characteristics and complications

	**Preoperative $R^2 = 0.037$**		**1 month $R^2 = 0.168$**		**12 months $R^2 = 0.301$**	
**Variable**	**Coef**	**p**	**Coef**	**p**	**Coef**	**p**
C30 global QoL, preop	-	-	0.27	<0.001	0.43	<0.001
Patient characteristics and operative data						
ASA classification	-6.0	0.020	-2.3	0.412	-6.0	0.012
Tumour stage	-4.6	0.042	NS	NS	NS	NS
Age	NS	NS	0.03	0.814	-0.29	0.007
BMI	NS	NS	NS	NS	-0.47	0.047
Preoperative radiation	NS	NS	-6.9	0.010	NS	NS
Preoperative chemo	4.4	0.129	NS	NS	NS	NS
Type of resection	-	-	PME: 0<br>TME: -1.3<br>APE: -3.7	0.693	NS	NS
Blood loss	-	-	-0.001	0.698	0.001	0.445
Length of hospital stay	-	-	-0.48	<0.001	-0.19	0.102
Skin-to-skin time	-	-	-0.002	0.874	0.000	0.984
			**1 month $R^2 = 0.175$**		**12 months $R^2 = 0.265$**	
**Variable**			**Coef**	**p**	**Coef**	**p**
C30 global QoL, preop			0.28	<0.001	0.39	<0.001
Postop complications						

Any	-3.7	0.500	4.9	0.223
Anastomotic leakage	-6.9	0.293	NS	NS
Respiratory	NS	NS	-14.9	0.012
Abscess	-2.2	0.750	NS	NS
Ileus	-16.9	0.029	NS	NS
Other	-1.7	0.750	-3.7	0.337
Re-intervention	-12.1	0.007	-9.0	0.013
Complications, 1 year				
Any	-	-	-1.4	0.503
Readmission	-	-	-2.6	0.189
Recurrence	-	-	-8.5	0.006

ASA classification was dichotomised into 1 vs. 2-4. Tumour stage was dichotomised into 1-2 vs. 3-4. NS corresponds to p>0.2 in bivariate analysis.

APE=abdominoperineal excision, ASA=American Society of Anaesthesiologists, BMI=body mass index, NS=non-significant, PME=partial mesorectal excision, QoL=quality of life, TME=total mesorectal excision.

Supplement Table 2. Multivariate models. Global HRQoL versus functioning domains, symptom scales and baseline global QoL in the EORTC QLQ-C30 questionnaire

	**Preoperative $R^2 = 0.545$**		**1 month $R^2 = 0.648$**		**12 months $R^2 = 0.603$**	
**Variable**	**Coef**	**p**	**Coef**	**p**	**Coef**	**p**
Baseline global QoL	-	-	0.08	0.058	0.15	0.000
Physical	0.22	0.003	0.01	>0.20	0.06	0.437
Role	0.02	>0.20	0.07	0.118	0.09	0.088
Emotional	0.23	0.000	0.13	0.019	0.11	0.052
Cognitive	0.01	>0.20	0.09	0.081	0.06	>0.20
Social	0.07	0.196	0.09	0.038	0.14	0.003
Fatigue	-0.13	0.033	-0.19	0.001	-0.19	0.004
Nausea and vomiting	-0.03	>0.2	-0.19	0.003	0.03	>0.20
Pain	-0.10	0.039	-0.10	0.007	-0.15	0.007
Dyspnoea	-0.03	>0.20	0.01	>0.20	-0.04	>0.20
Insomnia	-0.09	0.009	-0.01	>0.20	-0.02	>0.20
Appetite loss	-0.03	>0.20	-0.09	0.006	-0.10	0.082
Constipation	-0.02	>0.20	0.00	>0.20	0.01	>0.20
Diarrhoea	-0.08	0.003	0.03	>0.20	-0.01	>0.20
Financial difficulties	0.02	>0.20	0.04	>0.20	0.04	>0.20

HRQoL=health related quality of life.

Supplement Table 3. Multivariate models. Global HRQoL versus EORTC QLQ-CR38 domains and scales

	**Preoperative $R^2 = 0.425$**		**1 month $R^2 = 0.546$**		**12 months $R^2 = 0.424$**	
**Variable**	**Coef**	**p**	**Coef**	**p**	**Coef**	**p**
Baseline global QoL	-	-	0.27	0.059	0.23	0.004
Body image	0.19	0.051	-0.02	0.868	0.08	0.272
Sexual function	0.01	0.861	0.09	0.442	0.23	0.002
Sexual enjoyment	0.03	0.493	-0.09	0.390	-0.09	0.060
Future perspective	0.27	0.000	0.17	0.109	0.03	0.602
Micturition symptoms	-0.24	0.006	0.01	0.950	-0.08	0.403
Chemotherapy side effects	-0.09	0.463	-0.14	0.653	-0.31	0.038
Symptoms from GI-tract	-0.23	0.065	-0.03	0.854	-0.30	0.033
Male sexual problems	NS	NS	0.03	0.692	NS	NS
Female sexual problems	NS	NS	NS	NS	NS	NS
Defecation or stoma-related problems$^{\alpha}$	*	*	-0.13	0.472	0.03	0.668
Weight loss	-0.10	0.124	-0.05	0.636	-0.22	0.010

NS corresponds to p>0.2 in bivariate analysis.
*Only 6 patients had a stoma preoperatively, hence this analysis was excluded preoperatively.
$^{\alpha}$At 1 and 12 months, defecation and stoma-related problems were merged into a new variable.

GI=gastrointestinal, NS=non-significant, HRQoL=health related quality of life.
